# Low-cost luminescence lifetime system for medical diagnostics

**DOI:** 10.1088/2515-7647/ae15cf

**Published:** 2025-11-04

**Authors:** Lakhvir Singh, Melody Yeh, Linghao Hu, Brian Ko, Michael J McShane, Alex J Walsh

**Affiliations:** 1 Department of Biomedical Engineering, Texas A & M University, College Station, TX, United States of America; 2 Department of Material Science & Engineering, Texas A & M University, College Station, TX, United States of America; 3 Center for Remote Health Technologies & Systems, Texas Engineering Experiment Station, College Station, TX, United States of America

**Keywords:** fluorescence lifetime imaging, machine learning, phosphorescence lifetime imaging, low cost diagnostics, frequency domain fluorescence lifetime imaging, fluorescence, phosphorescence

## Abstract

Luminescence lifetimes provide insights into the local microenvironment of luminescent molecules, enabling precise measurements that can be leveraged for biomedical imaging and sensing applications. Traditional systems for measuring luminescence lifetimes rely on expensive ultrafast detectors and electronics. Here, a novel optical luminescence lifetime estimation method, modular analysis and eXtraction system for affordable lifetime imaging of fluorescence signals (MAX-alif), was developed to provide a high-speed, low-cost, and scalable solution for luminescence lifetime measurements. In MAX-alif, excitation light with sinusoidal intensity modulation is combined with synthetic path-length difference analysis and machine learning regression to compute luminescence lifetimes from intensity image features. An acousto-optical modulator within the detection path transfers the synthesized phase difference between the excitation and emission light into intensity differences that a standard camera can capture. Both simulation and experimental data were used to generate and validate models for the calibration and extraction of lifetime values from intensity datasets. MAX-alif measurements of phosphorescent and fluorescent samples achieved mean and median lifetime values within 5% of the reference lifetime values. By eliminating the need for expensive hardware and enabling accurate lifetime imaging with standard cameras, MAX-alif allows widespread, cost-effective luminescence lifetime imaging for medical diagnostics applications.

## 1. Introduction

Luminescence lifetime, the average time a luminophore remains in the excited state, reflects its molecular structure and is influenced by quenching and local environmental conditions such as temperature, pH, polarity, and mechanical forces [1]. Unlike intensity measurements, lifetime measurements are generally independent of luminophore concentration (apart from specific cases such as collisional self-quenching or aggregate formation [2]) and are minimally influenced by the optical properties of the surrounding medium or experimental factors like laser power or detector gain [1, 3].

In fluorescence lifetime imaging (FLIM), contrast arises from molecular decay dynamics, and the high sensitivity of FLIM has potential for both label-free functional imaging of endogenous fluorophores and targeted measurements with exogenous probes. For example, FLIM of endogenous fluorophores such as NADH, FAD, tryptophan, and porphyrins distinguishes malignant from healthy tissue by capturing shifts in fluorescence decay linked to altered metabolism [4-7]. Similarly, NAD(P)H and FAD lifetime signatures enable stem cell characterization and immune cell profiling, with machine learning further enhancing diagnostic utility [8-10]. FLIM with exogenous dyes allows quantification of pH, ion concentrations, and FRET interactions [11-13]. Targeted probes improve contrast in malignancies, enabling site-specific imaging with molecular precision [14]. Multiphoton FLIM visualizes skin lesions and inflammation at depth [15, 16], while intraoperative FLIM aids glioma delineation and liver disease monitoring [17, 18]. FLIM has also demonstrated sensitivity to detect progression of oral, vascular, and retinal diseases [19, 20]. As imaging speed and resolution improve, FLIM is poised for broader adoption in clinical diagnostics.

Similarly, phosphorescence lifetime imaging (PLIM) leverages the long-lived emission of phosphorescent probes, often based on iridium(III), platinum(II), or organic room-temperature phosphorescent materials, to assess biological microenvironments, particularly oxygen concentration [21-25]. These probes, functionalized for biocompatibility, exhibit oxygen-sensitive lifetimes that allow precise mapping of hypoxia, a key marker in many diseases including cancer [26, 27]. Phosphorescent dyes can also function as multianalyte biosensors, with oxygen- and glucose-sensitive lifetimes providing valuable readouts for monitoring diabetes, cardiovascular diseases, and microvascular complications [28, 29]. Compared to FLIM, PLIM offers longer emission lifetimes and reduced background autofluorescence, enhancing specificity and enabling deeper, high-contrast functional imaging *in vivo* [23]. Despite the promise of luminescence lifetime imaging for biomedical diagnostics, clinical adoption has been limited by the high cost and complexity of the measurement hardware. Luminescence lifetime measurements can be acquired by time-domain (TD) systems that measure the decay in intensity as a function of time from an excitation pulse, or by frequency-domain (FD) systems that measure the lifetime-induced phase shift between the fluorescence emission and intensity-modulated excitation light [30-33]. For TD laser scanning microscopy systems, luminescence lifetimes are acquired with single-photon detection and time-correlated single-photon counting (TCSPC) electronics to build histograms of photon arrival times for fitting and extraction of lifetime values [34]. Early demonstrations of lifetime imaging have established its diagnostic potential, and ongoing innovations in acquisition and analysis methods are enabling clinical integration [3, 17, 30]. Wide-field integrations of lifetime measurements, including time-gating, pulsed sampling, and FD methods, can be faster than TCSPC [35, 36], but are limited by hardware sampling rates, low sensitivity, and low photon counts for weak luminophores [3, 34, 37-41]. Recent innovations in compressed sensing methods and improvements in the quantum efficiency and temporal responses of gated intensified charge-coupled device cameras and single-photon avalanche diode arrays have improved camera-based lifetime imaging [42-44]. Yet, the high cost of the hardware, system installation, maintenance, and operational expertise remains a significant barrier to widespread implementation of lifetime technologies, particularly in resource-constrained clinical environments.

With the advent of fast processors and the increased accessibility of computational resources, multiplexing of hardware and software offers tunable, low-cost and compact solutions in many sensing and imaging domains [45-47]. Here, a computational microscopy approach is deployed to leverage conventional, low-cost optical components and machine learning regression for FD luminescence lifetime measurements from microseconds to nanoseconds using a consistent hardware design. The modular analysis and eXtraction system for affordable lifetime imaging of fluorescence signals (MAX-alif), uses sinusoidally-modulated light for excitation and modulates the emission to effectively transfer the phase difference between excitation and emission light into intensity differences captured by a standard camera. Intensity differences are related to lifetime values through a path difference set-up and calibration procedure that uses statistical features extracted from image stacks to train machine learning regression. Once calibrated, the accuracy of MAX-alif measured lifetime values was determined for platinum meso-tetra(4-carboxylphenyl) porphine (PtP), a phosphorescent molecule with a lifetime ≈96 *μ*s, fluorescein, a fluorophore with a lifetime ≈3.17 ns, and 20 *μ*m fluorescent YG microbeads with a lifetime ≈2.45 ns. Overall, MAX-alif is a cost-effective and efficient solution for luminescence lifetime imaging, offering significant promise for clinical applications such as cancer diagnosis, tumor margin delineation, cellular metabolism monitoring, oxygen sensing, and evaluating drug responses.

## 2. Methods

### 2.1. Working principle of MAX-alif

The MAX-alif system translates the luminescence lifetime-induced phase shift of intensity-modulated excitation light to a camera-integrated intensity difference via modulation of emission by an acousto-optic modulator (AOM) (figure 1). Intensity values are correlated to lifetimes through a regression-generated lookup-table. There are three main ideas in MAX-alif:

- *Encoding of high-frequency phase shifts As time-integrated intensity differences*: Conceptually, MAX-alif leverages the amplitude difference that results when waves of equal frequency but different phase shifts are combined (figure 1(a)). However, due to the positive intensity offset of the excitation and emission light, integration of high-frequency intensity-modulated signals for a realistic camera exposure time would yield identical values. To overcome this problem, the signal is chopped or modulated at a frequency that is synchronized to the peaks of the illumination intensity. This effectively converts the phase difference between the illumination and emission intensity-modulated signals into an intensity difference that is retained when the signal is integrated over a realistic camera exposure. Practically, since optical choppers are limited to kilohertz speeds, an AOM is used to convert the phase information encoded within the high frequency signal ($I_{c}$, figure 1(a)) to a signal ($I_{\text{final},\text{chopped}}$, figure 1(a)).
- *Calibration via synthetic waveform analysis*: In FD-lifetime measurements, the finite lifetime of the sample luminescence causes a phase shift between the intensity-modulated excitation and emission. The lifetime of the sample, τ, can thus be computed from the phase shift, Δϕ via equation (1),
(1) $$\tau =\left(\frac{1}{2\pi \;f_{m}}\right)\tan\;(\Delta \phi )$$
  where $f_{m}$ is the frequency of the sinusoidal modulation of the excitation light. For waves of equal frequency (such as the excitation and emission light) traveling at speed ν, the phase difference between the waves, Δϕ, is related to a distance (Δx):
(2) $$\Delta \phi =\frac{2\pi \Delta x\;f_{m}}{\nu }.$$
  By combining equations (1) and (2), the lifetime can be related to Δx:
(3) $$\tau =\left(\frac{1}{2\pi f_{m}}\right)\tan\left(\frac{2\pi \;\Delta xf_{m}}{\nu }\right).$$
  Equation (3) allows the calibration of MAX-alif through measurements acquired for varied path-length distances, Δx, to simulate different lifetime values, τ.
- *Machine learning regression for lifetime computation*: Machine learning regression models find the error-optimized relationship between a target variable and the sample feature dataset. In MAX-alif, regression is used to relate the path-length difference-encoded intensity features and the modulation frequencies to the corresponding path-length difference-derived lifetimes (supervised targets computed from equation (3)).

### 2.2. Computation model of MAX-alif and simulations to optimize frequency parameters

#### 2.2.1. Computational model of MAX-alif

First, an empirical relationship between lifetime and intensity values was found, as a function of camera parameters and modulation frequencies. The intensity profile of the intensity modulated excitation signal ($I_{\text{exc}}$) is defined as,

(4) $$I_{\text{exc}}(I_{d_{1}},I_{a_{1}},\phi ,t)=I_{d_{1}}+I_{a_{1}}\;\sin\;(2\pi f_{\text{exc}}t+\phi )\;\;(\text{exciation})$$

where $I_{d_{1}}$ is a DC offset of the sinusoid, $I_{a_{1}}$ is the amplitude, $f_{\text{exc}}$ is the modulation frequency, and ϕ is the instantaneous phase of the modulated signal. The intensity modulated emission signal ($I_{\text{em}}$), has the same modulation frequency as $I_{\text{exc}}$ but with a phase difference, Δϕ=2πΔx∕λ, introduced by the lifetime and related to a distance, Δx (as previously described in equations (1)-(3)).


(5) $$I_{\text{em}}\left(I_{d_{2}},I_{a_{2}},\phi +\frac{2\pi \Delta xf_{m}}{\nu },t\right)=I_{d_{2}}+I_{a_{2}}sin\left(2\pi f_{\text{exc}}t+\phi +\frac{2\pi \;\Delta xf_{m}}{\nu }\right)\;\;(\text{emission}).$$

To convert the phase difference between $I_{\text{exc}}$ and $I_{\text{em}}$ to an amplitude difference, $I_{\text{exc}}(I_{d},I_{a},\phi ,t)$ and $I_{\text{em}}(I_{d_{2}},I_{a_{2}},\phi +\frac{2\pi \Delta xf_{m}}{\nu },t)$ are combined, with the combined signal, $I_{c}$, given as

(6) $$I_{c}=I_{\text{exc}}\;(I_{d},I_{a},\phi ,t)+I_{\text{em}}\left(I_{d_{2}},I_{a_{2}},\phi +\frac{2\pi \Delta xf_{m}}{\nu },t\right)$$

The AOM provides an additional sinusoidal modulation at frequency $f_{\text{chopper}}$ to the combined intensity signal, $I_{c}$,

(7) $$I_{\text{final},\text{chopped}}=I_{c}\sin\left(2\pi f_{\text{chopper}}t\right)$$

$I_{\text{final},\text{chopped}}$ is then captured by a camera as $I_{\text{camera},\text{chopped}}$:

(8) $$I_{\text{camera},\text{chopped}}=\sum^{1∕\text{FPS}}\int_{0}^{T_{\text{exp}}}I_{\text{final},\text{chopped}}dt$$

where $T_{\text{exp}}$ is the camera integration time and $I_{\text{camera},\text{chopped}}$ is the signal measured by the camera during each integration block for each pixel. In the absence of an AOM, the camera-integrated signal, $I_{\text{camera},\text{unchopped}}$, is estimated by

(9) $$I_{\text{camera},\text{unchopped}}=\sum^{1∕\text{FPS}}\int_{0}^{T_{\text{exp}}}I_{c}dt.$$

Equations (4) through (9) were used in Python to simulate camera-measured intensities for MAX-alif measured lifetimes with and without the AOM. All signals were synthesized for t=0−2s with 10^6^ samples. The DC offset values, $I_{d}$ and $I_{d_{2}}$ , of the excitation and emission signals were both set to 5 W m^−2^. The sinusoidal amplification components, $I_{a}$ and $I_{a_{2}}$, were set as 3 W m^−2^ and 1.5 W m^−2^, respectively, for the excitation and emission signals. Signals were generated for modulation frequencies, $f_{\text{exc}}$, of 1 kHz, 2 kHz, 3 kHz, 4 kHz, 5 kHz, 35 kHz, 70 kHz and 105 kHz. Simulations were repeated for detection modulation frequencies, $f_{\text{chopper}}$, varying from 20 MHz to 100 MHz at 10 MHz steps. Camera frame rates of 51, 101 and 151 FPS were used, at a constant exposure time of 5 ms.

#### 2.2.2. Optimization of frequency parameters

To avoid harmonic distortion of the doubly-modulated emission signals, error of MAX-alif measured intensity values for pairs of $f_{\text{exc}}$ and $f_{\text{chopper}}$ values were computed. The following optimization function, $Q_{\text{opt}}$, was computed for given $f_{\text{exc}}$ and $f_{\text{chopper}}$ values:

(10) $$Q_{\text{opt}}=|\int_{0}^{\Delta t}S_{\text{cam}}(t)\;dt-\int_{0}^{\Delta t}S_{\text{aom}}(t)\;dt|-|\int_{0}^{\Delta t}S_{\text{cam}}(t)\;dt-\int_{0}^{\Delta t}S(t)\;dt|$$

where $S_{\text{cam}}(t)$ represents the unchopped signal (the signal from equation (9) computed for each simulated frame capture at a fixed exposure of 5 ms and time duration 0 to 50 ms with 10^6^ samples), $S_{\text{aom}}(t)$ represents the signal chopped by AOM (equation (8)), and S(t) represents the ideal signal $I_{c}$ under the same sampling conditions but with no camera integration (equation (6)). The optimization function $Q_{\text{opt}}$ uses the area under the curve of the modulated intensities (calculated using Simpson’s Rule) to find the optimal modulation parameters. The $f_{\text{exc}}$ and $f_{\text{chopper}}$ pair with the minimum $Q_{\text{opt}}$ is the optimal response that provides the least harmonic noise in the lifetime computations.

### 2.3. Experimental setup

An experimental setup for MAX-alif was designed to replicate the computational model (figure 2). First, the system was calibrated for different excitation and detection modulation frequencies (figure 2(a)). For calibration, the experimental system included an intensity-modulated diode laser (Thorlabs M530L3) of wavelength 530 nm as a light source. The modulation of the diode laser was achieved by using a function generator to create a sine wave with 3 V peak-to-peak amplitude. For frequencies between 1 kHz and 5 kHz, a Texas Instruments NI myDAQ, controlled by NI ELVISmx software, was used. For excitation modulations of 35 kHz, 70 kHz, and 105 kHz, a Hewlett Packard 33 120A function generator was used. The modulated light was collimated by a series of lenses: a Plano-Concave Lens with 30.0 mm focal length (LC2679-A—∅1’, N-SF11), followed by an Achromatic Doublet with 150 mm focal length (AC254-150-A ∅1’), then an Achromatic Doublet lens with 30 mm focal length (AC254-030-A-ML ∅1’), and finally a Achromatic Doublet lens with 75 mm focal length (AC508-075-A ∅2’). This yielded a collimated beam with a 3 mm diameter.

The collimated beam then passed through a 60:40 polarizing beam splitter (PBS), which split the light into two different paths: 40% was directed towards a fixed mirror ($M_{1}$) set at a distance of 20.32 cm to create a reference arm and 60% to a translatable mirror ($M_{2}$), initially set at the same distance, for the calibration arm. The beams returning from mirrors $M_{1}$ and $M_{2}$ were combined by the PBS to create $I_{c}$, which then passed through an AOM (IntraAction AOM-802 R). The AOM has an aperture of about 5 mm × 4 mm and a center frequency of 80 MHz. The modulation of the AOM was controlled by a voltage-controlled oscillator (FeelElec FY6900) and amplified by an AOM RF power amplifier (IntraAction GE RF4030). The AOM was operated in the Bragg diffraction mode, with the modulation encoded in the first diffraction mode. The signal coming out of the AOM, $I_{\text{final},\text{chopped}}$, was imaged with a CMOS camera (ThorLabs CS165MU—Zelux^®^ 1.6 MP Monochrome) to measure $I_{\text{camera},\text{chopped}}$.

For collection of calibration data, $M_{2}$ was translated towards the PBS one step (10 *μ*m) at a time for ten iterations to simulate a lifetime-induced change in phase shift via path-length difference (figure 2(a)). For each path-length step of $M_{2}$, 200–400 image frames were acquired with a fixed frame rate of 34 frames per second (FPS). Image stacks were acquired for varying $f_{\text{exc}}$ and $f_{\text{chopper}}$ values. The $f_{\text{exc}}$ values were 1, 2, 3, 4, 5, 35, 70, and 105 kHz. For each $f_{\text{exc}}$, the $f_{\text{chopper}}$ values were varied between 20 MHz and 100 MHz at 10 MHz steps. For each pair of $f_{\text{exc}}$, the $f_{\text{chopper}}$ value, the path length was stepped from 10 *μ*m to 100 *μ*m in 10 *μ*m increments to complete the calibration. The calibration data was used to generate and test regression models to relate the camera-measured intensity features with phase differences for the lifetime calculation.

### 2.4. Measurement of phosphorescence and fluorescence lifetimes

Once calibrated, MAX-alif was evaluated by imaging the luminescence lifetime of PtP, fluorescein and 20 *μ*m YG microbeads. The samples were prepared in solution (supplemental information section 3). For imaging of luminescent samples, $M_{2}$ was replaced by a beam blocker and a sample was placed in the measurement arm (figure 2(b)). $M_{1}$ remained at the set distance of 20.32 cm. A 530 nm intensity-modulated (sinusoidal) excitation light source was passed through the collimation arm and PBS, then through the sample, which was held inside a soda-lime glass capillary tube of 1.5 mm ± 0.05 mm diameter. The diameter of the illumination beam was around 3 mm and the laser power at the source was ~ 65 mW at the sample (after absorption loss in the relay optics). The detection arm after the sample included high NA (5.0) lenses of focal lengths of 75 mm and 100 mm, respectively, which focused the light from the sample into the AOM. The doubly-modulated light was then focused through another pair of lenses with focal lengths of 25 mm and 50 mm, respectively. The focused light was filtered to attenuate the excitation light using a 600 nm ± 40 nm bandpass filter (Thorlabs FBH00-40) and imaged by the CMOS camera. Fluorescein was imaged using camera integration times of 15 ms (34 FPS) and 29 ms (34 FPS), while PtP was imaged using 55 ms (18 FPS) and 95 ms (10 FPS) frame integration times. A reflectance configuration was also developed for MAX-alif as described in supplemental information section 5, and used to image fluorescein solutions and 20 *μ*m YG microbeads.

The ground truth value of PtP was measured with a PTI QuantaMaster spectrofluorometer and the phosphorescence intensity vs. time curve was fit to an exponential decay model (section 4, supplemental information). Different exclusion fractions were used to trim data points from the beginning and end of the intensity-time plot to remove noise from the dataset and obtain better fits, as determined by reduced *χ*^2^ values. The lifetime values for fits with reduced *χ*^2^ less than 10 were used to find the mean and standard deviation of the lifetime.

A two-photon FLIM system with TCSPC electronics, as previously described [48], was used to acquire ground truth fluorescence lifetime images of the fluorescein solution. The fluorescence lifetime was imaged with the tunable laser (Chameleon Ultra II, Coherent) set to an excitation wavelength of 1000 nm, an intensity power of 8–15 mW, and a 550 nm ± 88 nm emission filter. The ground truth values for the YG microspheres were also taken using the two-photon FLIM system with an excitation wavelength of 890 nm (5–10 mW). The mean lifetime values for the two-photon FLIM images were calculated by a single-exponential decay fit in the SPCImage software.

### 2.5. Image processing and lifetime estimation

#### 2.5.1. Calibration

The calibration data was used to generate the regression models that decipher synthetic phase shifts and compute lifetimes. First, the calibration data underwent pre-processing, training, and computation steps. In the pre-processing step, an intensity-based threshold was applied to the image stack to exclude pixels that were either saturated or contained insufficient photons. Specifically, pixels with intensities below 2.75% or above 78.90% of the maximum intensity were removed for each frame. The resulting image stack was the input to a customized processing code (Python), which extracted statistical values of mean, standard deviation, skewness, and kurtosis frame-wise (figure 3). The code also combined the statistical values of each image stack with the corresponding modulation frequencies $f_{\text{exc}}$ and $f_{\text{chopper}}$ and the lifetime value corresponding to the path length calculated from equation (3). The data was saved in a .CSV (comma separated values) file with the extracted feature values arranged in columns.

Next, the calibration data was used to train the regression model to relate intensity features and lifetimes. The feature data was segregated by emission modulation frequency, $f_{\text{chopper}}$. XGBoost random forest regressor was implemented in Python to predict the lifetime values from input features (mean intensity, standard deviation, skewness, and kurtosis) for each emission modulation frequency. The XGBoost random forest regressor used a squared error loss objective function, a learning rate of 0.01, 6000 boosting rounds, a maximum tree depth of 5, fraction of features to be randomly sampled for each tree as 90%, and L1 regularization term on weights as 1. The models that gave an *R*^2^ score of 0.8 and greater for each $f_{\text{chopper}}$ frequency were then averaged and stored as a pickle file (a binary file that stores serialized Python objects) to represent the final calibrated model.

#### 2.5.2. Computing lifetime of samples

Computation of the sample lifetimes followed a similar process as the analysis of the calibration data. Firstly, pixels with intensities below 0.8% of the maximum intensity were set as NaN (not a number). The intensity features, mean, standard deviation, skewness, and kurtosis, were calculated for each pixel across the image stack. The pickle file of the calibrated model was imported and used to compute the lifetime value for each pixel of the sample image. An upper threshold of 2000 *μ*s and lower threshold of 0 *μ*s were set to confine the lifetime values. A reference image stack that corresponded to the zero path-length difference for iteration of $M_{2}$ mirror was used for pixel-wise lifetime computation. The final lifetime was computed after taking an $L_{1}$ norm of the sample with respect to the reference dark noise image, as shown in equation (11).


(11) $$\tau_{\text{final}}={||\tau_{\text{sample}}-\tau_{\text{ref}}||}_{1}$$

## 3. Results

### 3.1. Simulation results

Simulations of the computational model described by equations (1)-(9) explore the principles underlying MAX-alif. The phase shift between the modulated emission signal, $I_{\text{final},\text{chopped}}$, and $I_{\text{exc}}$ exhibits a relationship with the normalized mean of $I_{\text{camera},\text{chopped}}$. For simulations of $I_{\text{camera},\text{unchopped}}$, the peak mean intensity fluctuates with phase shifts from 81 degrees to 180 degrees and is inconsistent for different frame rates and modulation frequencies (figure 4(a), table S1). In contrast, the chopped signal, ($I_{\text{camera},\text{chopped}}$), maintains a consistent peak mean intensity, curve uniformity, and symmetry around the 90-degree peak with a standard deviation of less than 1 degree (figure 4(b), table S1). This symmetry is consistent for higher frame rates (101 and 151 FPS) across modulation frequencies (table S1).

The simulations of camera-integrated intensity demonstrate that the $I_{\text{camera},\text{unchopped}}$ signal yields identical values for different phase shifts between excitation and emission signals (figure 4(c)). Chopping or modulating the signal at a frequency greater than or equal to the excitation modulation frequency results in different intensity values for different phase shifts (figure 4(d)). Higher phase shifts show reduced intensities for $I_{\text{camera},\text{chopped}}$ as hypothesized (figure 4(d)), figure 1(a)).

#### 3.1.1. Frequency optimization

Computation of the optimization function (equation (10)) for the MAX-alif system allows for analysis of modulation frequencies of the excitation light and detection arm. For each $f_{\text{exc}}$, the optimization function-based error values ($Q_{\text{opt}}$) decrease as $f_{\text{chopper}}$ increases (figures 4(e) and (f)). Conversely, for a constant detection modulation frequency, $f_{\text{chopper}}$, the error values generally decrease for increased $f_{\text{exc}}$ for frequencies in the MHz range (figure 4(e)). For $f_{\text{exc}}$ in the kHz range figure 4(f), the error values are consistent across different $f_{\text{chopper}}$ values. The optimal frequency pairs for imaging were determined from the error values, with $f_{\text{exc}}$ and $f_{\text{chopper}}$ set to 70 kHz and 80 MHz for the phosphorescence regime. For phosphorescence frequencies, overfitting introduced increased errors for higher $f_{\text{chopper}}$ values (figures S1(d)-(f)). For MHz range frequencies (for typical fluorescence regime), the optimal frequency pair for $f_{\text{exc}}$ and $f_{\text{chopper}}$ was 80 MHz and 90 MHz, respectively, for 51 FPS. For higher FPS, the optimal frequency pair was 50 MHz and 90 MHz, respectively. An improved error-optimized response was observed for frequency pairs with $f_{\text{chopper}}$ close to double $f_{\text{exc}}$ (figures S1(a)-(c)).

#### 3.1.2. Linearity of path-length distance derived lifetimes

Simulations of equation (3) were performed to define the relationship between Δx and τ. For Δx values between 0 *μ*m and 9 *μ*m, the relationship between Δx and lifetime, τ, is linear with a linearity score (*R*^2^) exceeding 0.99 (figure 5(a)). Δx values of 0.1 *μ*m and 10 *μ*m correspond to lifetime values of 3.33 mfs and 33.33 fs, respectively. Nanosecond lifetimes correspond to Δx values of 0.1–0.6 m (figure 5(b)). For nanosecond lifetime values, a linear relationship between Δx and τ is maintained for excitation modulation frequencies, $f_{\text{exc}}$, of 1 and 10 MHz (figure 5(b)). Slight nonlinearity is observed between Δx and τ for $f_{\text{exc}}$ of 50 MHz, and an exponential relationship is observed between Δx and τ for 100 MHz $f_{\text{exc}}$ (figure 5(b)).

The high linearity of the *μ*-range relation between Δx and τ allows for scaling of the path length range needed for computing realistic nanosecond or microsecond lifetime values by scaling of the supervised targets, the path difference-based lifetimes obtained from equation (3). A scaling factor of 3.5 × 10^13^ for equation (3) allows computations of lifetime values ranging from 1 ns to 11 ns with an associated Δx that varies from 0 to 100 *μ*m, and scaling by 1.2 × 10^15^ allows computations of lifetimes of 40 *μ*s to 360 *μ*s for the same path length range.

### 3.2. Experimental results

#### 3.2.1. MAX-alif model calibration

Calibration experiments were conducted by translating a mirror in 10 *μ*m steps to create path-length differences for generating training data of the MAX-alif XGBoost-based regression fits to compute lifetimes. The optimized MAX-alif regression models have root mean square errors of 0.5 ns for fluorescence lifetimes (figure 5(c)) and 15.4 *μ*s for phosphorescence lifetimes (figure 5(d)). The relationship between the model-computed and the path length analysis-derived reference lifetimes have *R*^2^ scores of 0.97. Feature analysis of the models revealed a dependence on mean intensity (μ) (46.8% for fluorescence and 50.6% for phosphorescence regime), followed by skewness ($\gamma_{1}$) (26.4% and 20.7%, respectively), and then standard deviation (σ) (16.0% and 18.6%, respectively)(figures 5(c) and (d)). The dependence of kurtosis (κ) was 8.6% and 7.9%, respectively, and the dependence on modulation frequency was minor (<3%).

#### 3.2.2. MAX-alif measurements of fluorescence lifetime

The MAX-alif lifetime distributions for fluorescein showed a stable lifetime response at different exposure times. For a camera integration time of 15 ms, the MAX-alif measured lifetime of fluorescein was normally distributed with a mean of 3.00 ns ±0.07 ns (figures 6(a)-(i)). For a camera integration time of 29 ms, the MAX-alif measured lifetime value of fluorescein was similar, with a mean of 3.02 ns ± 0.05 ns (figures 6(a)-(ii)). The MAX-alif measured fluorescence lifetime of fluorescein was consistent at 3 ns, within the standard deviation of TCSPC-measured value of 3.17 ns ± 0.18 ns, as long as the statistical features were extracted from intensity image stacks consisting of at least 6 frames, for both 15 ms and 29 ms at a constant frame rate of 34 Hz (figure 6(b)).

#### 3.2.3. MAX-alif measurements of PtP phosphorescence lifetime

The mean values of lifetimes for the phosphorescent sample of PtP were constant for different camera integration times. For 55 ms exposure, the MAX-alif measured lifetime of PtP was normally distributed with a mean of 97.87 *μ*s and standard deviation of 8.47 *μ*s (figure 6(c)-(i)). For 95 ms exposure, the PtP lifetime was 96.6 *μ*s ± 4.8 *μ*s (figure 6(c)-(ii)). The MAX-alif measured PtP lifetime values were within the standard deviation of the spectrofluorometer measured value, 95.77 *μ*s ± 6.13 *μ*s (figure S4). When MAX-alif PtP lifetime measurements were evaluated for temporal stability, at least 400 frames were required for the convergence of MAX-alif lifetime values with the spectrofluorometer-measured value of 95.77 *μ*s ± 6.13 *μ*s (figure 6(d)). The results were consistent for different camera integration times used for capturing time-varying image stacks (55 ms/18 FPS and 95 ms/10 FPS).

#### 3.2.4. MAX-alif measurements in reflectance mode

MAX-alif was also demonstrated in a reflectance configuration. To evaluate lateral resolution, a USAF-1951 target was imaged with the Olympus 10×/0.25 NA objective (2.27 *μ*m/px). A line profile across the bar pattern was analyzed to generate the edge spread function and the line spread function (LSF) (figure S5 of supplemental information). The full-width at half maximum (FWHM) of the LSF was measured to be approximately 18 *μ*m, which represents the effective spatial resolution of the setup. The temporal calibration of the system was performed by selecting the optimal parameters for iterative calibration parameters. The excitation arm and the emission arm modulation frequencies were chosen from the table (figure S6(a)) generated by optimizing the function given by equation 10. The optimal value for both excitation and AOM modulation frequencies was 90 MHz. The resulting calibration fit provides a high linearity score of 0.83 and a minimal root mean square error of 0.82 ns (figure S6(b)) for the range of lifetime values from 1 ns to 11 ns. The feature contribution analysis showed a high dependence on mean intensity with a score of 53.1%, followed by standard deviation at 35%, kurtosis at 6.5%, and skewness at 5.4% (figure S6(c)).

The FLIM capabilities of MAX-alif allow visualization and quantification of fluorescein and 20 *μ*m YG bead fluorescence (figures 7(a)). MAX-alif resolved the YG microsphere lifetimes and fluorescence intensity successfully against the background (figures 7(a)-(v) and (a)-(vi)) and provides lifetime values for fluorescein and YG beads (figures 7(a)-(iii) and (a)-(vi)). The distributions of MAX-alif lifetime values across image pixels for both samples have high overlap with the lifetime distribution measured from TCSPC-FLIM (figures 7(b) and (c)). The MAX-alif computed lifetime values determined from image stacks of different numbers of frames converge after 7 frames for both the fluorescein and YG beads (figure 7(d)) and are within 3% of the reference value for fluorescein (3.17 ns ± 0.18 ns) and within 1% of the TCSPC-FLIM value for the YG beads (2.45 ns ± 0.28 ns).

## 4. Discussion

The MAX-alif system is a computational imaging approach that leverages wave properties of intensity-modulated excitation light, experimental path difference analysis, and machine learning regression to provide a rapid luminescence imaging system, while avoiding the use of expensive instruments. Here, both simulations and experiments provide a complementary foundation to prove the concepts underlying MAX-alif and demonstrate its accuracy and consistency for measuring fluorescence and phosphorescence lifetimes. MAX-alif successfully used path difference induced phase shifts of intensity-modulated light to experimentally simulate different lifetime measurements to acquire training data for a lifetime regression model. The machine learning regression-based look-up table then reported lifetimes of PtP, fluorescein, and YG microbeads within 5% of the values measured by gold standard systems (figure 6), demonstrating the robustness and breadth of applicability of the system for both PLIM and FLIM.

In imaging systems like computer generated holography or optical coherence tomography, an interference pattern between the reference beam and object beam is used to extract sample information like the depth profile or the quantitative phase [49, 50]. In MAX-alif, waveform path difference analysis is leveraged for sinusoidal intensity modulated signal and is not optical interferometry. The synthetic path difference of the intensity-modulated light computationally represents the contrast in intensity at different path lengths of the calibration arm. The intensity profile of light with a reference path length compared to one with an increased path length mimics the delay caused by a phase shift, such as those induced by luminescence lifetime. Thus different lifetime-encoded intensity signatures of physical path lengths can be recorded as supervised targets for machine learning regressors to compute the lifetime from sample intensity.

Modeling and analysis confirmed the concepts of MAX-alif, showing that the intensity of sinusoidally modulated excitation light varies with different lengths of the detection arm, resulting in distinct phase differences in the intensity-modulation waves of the excitation and detection light (figure 4). This phase shift is correlated to a lifetime value as is defined for traditional FD-FLIM (equation (1)) [39]. For detection of the phase shift in MAX-alif, an AOM is used to encode phase information into intensity differences (figure 4(d)). Simulations confirm that double modulation conserves lifetime information (figure 4 and table S1 of supplemental information). Validation experiments demonstrate the linear relationship between computed lifetimes and the supervised targets (*R*^2^ score for testing > 0.8) for measurement and reference arms having same (figures 4(c), (d) and figure S2) and different optical wavelengths (figure S3). The regression models used here are dependent on the calibration approach based on experimental path difference analysis. Unlike traditional FD-FLIM systems that depend heavily on the synchronization of the modulated light source and phase-sensitive detection electronic through same-session calibration with reference signals, MAX-alif does not depend on complex synchronization between the light source and detector, and is robust for an initial calibration that only needs to be repeated if the hardware is altered [38, 51-53]. By leveraging regression models calibrated through path difference analysis, MAX-alif achieves accurate lifetime measurements without relying on external modulation references, making it more adaptable to diverse experimental setups, reflecting on its robustness, flexibility, and simplification of the system architecture.

Traditional lifetime imaging systems face challenges in performing both FLIM and PLIM due to the distinct temporal ranges of fluorescence and phosphorescence lifetimes. FLIM is optimized for nanosecond-scale lifetimes and utilizes high-speed timing electronics such as TCSPC modules or FD modulation in the MHz–GHz range to do so. For PLIM, lower modulation frequencies or delayed gating are typically required to resolve the slower decay kinetics, which are typically incompatible with the high-frequency electronics optimized for fluorescence. Furthermore, the analysis models used for lifetime extraction are often optimized for a specific timescale, limiting their ability to simultaneously accommodate both short and long lifetimes [3]. Addressing these challenges typically requires system modifications, such as integrating broader dynamic range detectors, multi-frequency modulation capabilities, and advanced signal processing algorithms, all of which are cost and computationally intensive [34, 54]. In the phosphorescence regime, smartphone-based lifetime imaging techniques that utilize time-gated acquisition or the rolling shutter mechanism of the camera have been demonstrated to capture decay dynamics [29, 55, 56]. However, a low-cost luminescence solution capable of performing both FLIM and PLIM measurements on the same platform has yet to be demonstrated. In MAX-alif, the same hardware setup can be used for MAX-alif measurements of fluorescence or phosphorescence lifetimes, with the different ranges achieved simply by tuning the modulation frequency and selecting the appropriate regression model (figures 5(c) and (d)).

The achievable range and precision of measurable lifetime values are fundamentally determined by the path length range, path length resolution, modulation frequency, and the regression model. Since the instrument has an operational range limited by these factors, the lifetime regime of the sample, either fluorescence or phosphorescence, is important to know. Reasonably, the system can be calibrated to measure fluorescence lifetimes between 0.1 ns and 8 ns, and phosphorescence lifetimes between 40 *μ*s and 300 *μ*s. The range of lifetime acquisition for MAX-alif depends on the calibration dataset, with a broader dataset with more iterations of path length and modulation frequencies producing a broader range of computed lifetimes at increased accuracy.

Practically, the lifetime resolution and precision can be estimated from the experimental results and RMSE of the regression models. For the reflectance configuration, MAX-alif was able to detect YG beads with a lifetime of ~2.5 ns and fluorescein with a lifetime ~3 ns, suggesting an effective lifetime resolution of at least 0.5 ns (figure 7(d)). The lifetime values reported here are mean lifetime values, computed across pixels, yet the calibration profile adds an additional standard deviation or potential error to the lifetime computation that is not directly accounted for in the mean lifetime calculations (figure 7(d)). For samples within 2–5 ns, MAX-alif has a low RMSE error of 0.22 ns between the reference and computed lifetimes, and higher RMSE values of 0.82 ns for lifetimes 6–7 ns (figure S6). From the RMSE of the regression model, a practical resolution can be estimated as 2xRMSE, which would be ~0.5 ns for fluorescence lifetimes between 2–5 ns and ~1.5 ns between 6–7 ns, and for phosphorescence lifetimes it would be ~30 *μ*s between 40 *μ*s and 300 *μ*s.

In MAX-alif, fluorescence lifetime is extracted from phase-encoded intensity values by leveraging the theoretical linear relationship between mean intensity and emission phase shift (figures 4(b), (d) and equation (1)). This relationship, along with the linear dependence between path length and lifetime (figures 5(a) and (b)), enables lifetime calibration via scalable path-length differences. For accurate lifetime output, FD-FLIM typically requires the frequency of excitation modulation to be close to or slightly higher than the inverse of 2*π* times the desired lifetime resolution [1, 57]. Therefore, high excitation modulations, >50 MHz, are required, but at these modulation frequencies, the path-length differences and lifetime relationship is non-linear (figure 5(b)). Low $f_{\text{exc}}$ (<10 MHz) have high linearity (figures 5(a) and (b)) but reduced ability to distinguish close or small (nanosecond) lifetime values. Furthermore, for MAX-alif, lower excitation modulation frequencies induce higher deviations between lifetimes for varying $f_{\text{chopper}}$ frequencies (figures 4(e) and (f) and the plots of mean intensity and phase shift in table S1). In the fluorescence regime, we observed that the simulated unchopped camera response deviated more strongly from the ideal case at modulation frequencies of 10, 30, 50, and 70 MHz, whereas closer agreement was obtained at 20, 40, and 60 MHz (table S1). These deviations are attributable to aliasing and phase sampling effects within the simulation rather than inherent hardware constraints. In addition, the chopped-intensity simulations produced irregular results at 1 FPS, which can be explained by undersampling and reduced temporal averaging at this low frame-rate. While these artifacts do not affect the general validity of the method, they highlight specific parameter ranges where care must be taken in interpreting simulation outputs. Therefore, higher $f_{\text{exc}}$ and high $f_{\text{chopper}}$ will give the best response for fluorescence lifetimes.

MAX-alif overcomes the high non-linearity between path length and lifetime for high modulation frequencies by using short, *μ*m, path-length differences for calibration followed by scaling factors. Furthermore, MAX-alif utilized an LED source with a maximum modulation frequency limited to kilohertz so the sample imaging was performed at $f_{\text{exc}}$ of 70 kHz for both fluorescein and PtP with an $f_{\text{chopper}}$ of 80 MHz. Despite the limited excitation modulation frequency (which is lower than the optimal modulation frequency required for fluorescein), accuracy within 5% deviation of the mean gold standard lifetimes was obtained, showing the robustness of the approach. Future improvements in LED selection, particularly those capable of higher modulation frequencies, could significantly enhance the system temporal resolution and overall accuracy.

In MAX-alif, lifetime information is derived from phase difference encoded intensity values. However, the intensity changes due to small lifetime differences are hard to capture with a conventional camera due to limitations of the camera capture speed, sensitivity, and exposure parameters. The practical limitations of conventional cameras require a novel approach to decode minute intensity shifts. Advanced signal processing and machine learning models have been demonstrated to computationally improve the information captured for conventional cameras [47, 49, 58]. Similarly, in MAX-alif, multiple statistical features of the measured intensity signal are used within the regression model. Statistical features such as standard deviation highlight local heterogeneity, spatial and temporal variations, and high-frequency components of the signal [59]. In MAX-alif, the inclusion of the statistical features of intensity, along with the mean, provides increased accuracy for lifetime computation and a more precise regression analysis. Prior works within the fields of biomedical engineering, astrophysics, and computational neuroscience have demonstrated the usefulness of including statistical features in optical and phase imaging [59-61]. For example, standard deviation captures heterogeneity within radiation-induced morphological changes in blood cells [59]. Notably, for calibration of MAX-alif, the importance of mean intensity was weighted 50% (figure 5), a large proportion as expected due to the theoretically linear relationship between lifetime and mean intensity. Furthermore, the importance of mean intensity as a feature for regression fit is highest for the case of AOM frequencies between 70 MHz and 90 MHz (figure S2 and S3 in supplementary material), validating the frequency optimization model for MAX-alif (equation (10), figures 4(e), (f) and S1).

The clinical value of FLIM and PLIM lies not only in their molecular specificity and label-free contrast but also in their ability to deliver rapid, nondestructive assessments of tissue function. For instance, in cancer imaging, the speed of lifetime measurements is essential for intraoperative decision-making, where real-time feedback can delineate tumor margins and preserve healthy tissue. As lifetime-based imaging continues to evolve, tailoring its temporal resolution to match the demands of each application will be critical in translating these technologies from bench to bedside. Traditional FLIM systems such as laser-scanning TCSPC require imaging times of tens of seconds to minutes [3] to collect an image and traditional curve-fitting algorithms are slow to an iterative least-squares fitting process. Machine learning has emerged as a useful tool for rapid analysis of raw TD FLIM data instead of performing iterative exponential decay fitting for each pixel [45, 62, 63]. Approaches such as artificial neural networks (ANNs) and the expectation–maximization algorithm have been effectively employed for efficient lifetime estimation [62, 63]. ANN-based fitting can achieve speeds up to 180 times faster than conventional least-squares methods [62]. To address the computational and memory challenges posed by large-scale spatio-temporal training data, compressive sensing strategies such as those introduced by Yao *et al* have been implemented to reduce data dimensionality while preserving reconstruction accuracy [64, 65]. Leveraging a simplified, fast and explainable machine learning regression (XGBoost) for calibration and prediction, MAX-alif can quantify lifetimes at high imaging speeds. The frame rate optimization for both the samples of PtP dye and fluorescein showed the stability of lifetime response at 400, and 7 frames respectively (figure 6), which equates to 4.86 Hz for fluorescence imaging and 0.045 Hz for phosphorescence imaging using a frame rate of 34 FPS and 18 FPS respectively. The slower convergence for phosphorescence is expected due to the slower nature of phosphorescence and weaker nature of the PtP dye [66]. A frame rate of 5–6 Hz is comparable to FD-FLIM and wide-field TD-FLIM, yet high speed FLIM systems face sensitivity and cost limitations due to the reliance on expensive, ultrafast detectors and electronics [3, 40, 67]. Wide-field TD-FLIM at 29 FPS has been reported, yet high-speed FLIM requires trade-offs between speed, sensitivity, and resolution [68]. Similarly, the compressed sensing approach for FD-FLIM allows lifetime imaging at 100 FPS using a streak camera, yet is limited by the temporal sequence depth and high cost of streak cameras [65]. The ability of MAX-alif to compute accurate lifetimes with reasonably rapid imaging speeds addresses some limitations of conventional, high cost FLIM systems and rivals real time luminescence lifetime imaging capabilities.

The spatial resolution of the MAX-alif system was demonstrated for the reflectance imaging setup. MAX-alif provides clear spatial contrast between the 20 *μ*m YG beads and background (figures 7(a)-(v) and (a)-(vi)). With 450 nm excitation and a 10×/0.25 NA objective, the diffraction-limited resolution is ~1.1 *μ*m, while the camera sampling of 2.27 *μ*m/px constrains detail to ~4–5 *μ*m. Additionally, the field of view of the image is restricted by the AOM aperture. In practice, the USAF-1951 target yielded an effective resolution (FWHM) of ~18 *μ*m (figure S5(c)). While this resolution is sufficient for wide-field lifetime imaging, it also indicates potential for optimization of the optical and detection pathway to further enhance spatial performance.

Traditional FD systems are limited by detector non-linearity and phase drift [69]. Artifact correction is necessary to ensure reliable quantification for informed diagnosis and treatment [16, 17]. The temporal stability of MAX-alif measured lifetimes are an advantage. MAX-alif lifetime measurements were independent of the image integration time (figure 6). While traditional high-cost PMT and other linear detectors have wider dynamic range of linearity than the scientific cameras (pixelated CMOS/CCD detectors), the wide-field image capture by the cameras reduces the phase drift that can occur during sequential capture, which is common in PMTs and other scanning mode detectors [1]. Although there is a photon flux limit imposed by pixel saturation, exposure times and illumination intensity can be optimized to minimize or eliminate saturation. The AOM used in the detector can introduce non-uniformities which are accounted for by the optimization of modulation frequencies in this work. All this ensures that MAX-alif measurements have low phase drift and high linearity.

Selection of MAX-alif components were driven by function and low-cost design. The cost of the LED laser, LED driver, function generator, and digital oscilloscope combined was around $900, the AOM and driver was around $600, and the combined cost of lenses and optical components was less than $300. The remaining cost is attributed to the camera, which is tunable to the needs of the imaging system. Here, a ThorLabs CS165MU - Zelux^®^ 1.6 MP Monochrome camera which costs around $ 515 was used for transmission mode. The final cost of the MAX-alif system could be estimated between $2100–$2500. For reflectance mode, Pixelink M-Series M12BM-CYL Microscopy Monochrome camera was used which costs around $3000, keeping the final cost still below $5000. This is a significant improvement over traditional low-cost FD and TD systems that cost at least tens of thousands of dollars [38, 70].

Overall, the MAX-alif system performs rapid luminescence lifetime image acquisition and processing with a highly accessible and low effort footprint. The processing time for image analysis of a single image stack with 400 frames on a computer with 2.90 GHz processing speed is between three to five minutes, including the time to manually provide the input parameters. Additionally, the tunability of this system allows for the adjustment of imaging parameters and optimization of speed, sensitivity, and resolution based on specific requirements, making it suitable for sensing dynamic chemical or biochemical environments. The ability to adjust these parameters ensures that imaging can be performed efficiently and within cost constraints, addressing some of the limitations inherent to conventional lifetime imaging technologies.

Further validation using well-characterized reference fluorophores under controlled environmental conditions would provide an additional benchmark for lifetime resolution and is a natural next step for this platform. In the present study, comparison with dyes of established lifetimes already demonstrated close agreement with reported values, supporting the reliability of the approach. Beyond these validations, the computational framework can be readily extended to multi-exponential lifetime analysis. Both regression- and phasor-based strategies are adaptable to bi-exponential and more complex decay models, enabling broader application to metabolic imaging. Such developments would allow quantification of the relative contributions of free and protein-bound NADH, or other short- and long-lived species, thereby enhancing the biological impact of this method. By providing an accessible starting point, the MAX-alif solution for lifetime imaging can accelerate the development of clinically deployable tools and enable methodical investigation of their diagnostic effectiveness given the high potential of fluorescence and phosphorescence lifetime as robust technologies for medical diagnostics.

## 5. Conclusion

We have demonstrated a low-cost optical luminescence lifetime measurement and imaging system that can accurately image lifetimes from microseconds to nanoseconds using a streamlined, compact, and cost-effective setup with a tunable computational framework. The system was validated on fluorescent samples of fluorescein and 20 *μ*m YG microbeads and a phosphorescent sample of PtP dye. In each case, the lifetime distributions were within 5% of gold standard lifetime values. Optimization of the frame counts used for lifetime computation showed a stable response after 6 frames and 400 frames for nanosecond and microsecond lifetimes, respectively. The rapid, cost-effective FLIM and PLIM system prototype presented here paves the way for advances in cellular and tissue health monitoring and facilitates broader integration of FLIM and PLIM into medical diagnostics.

## Supplementary Material

Supplementary Figures and Tables

Supplementary material for this article is available online

## Figures and Tables

**Figure 1.**
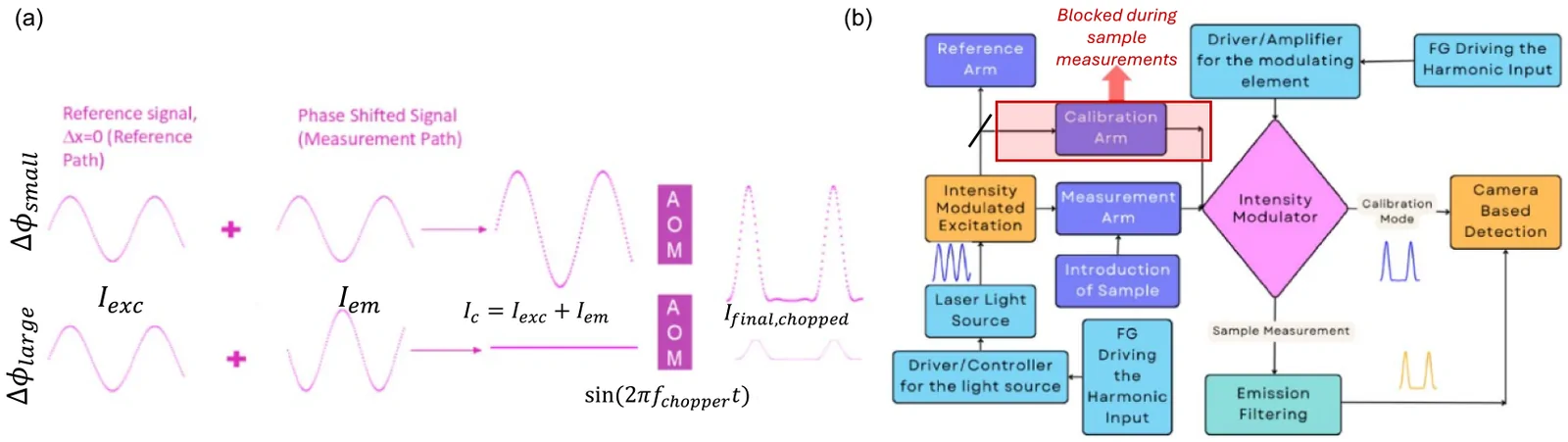
(a) Illustration of the theory of MAX-alif. The phase difference between excitation and emission intensity-modulated light is converted to a time-integrated intensity difference through an AOM. (b) Schematic of the MAX-alif system illustrating the implementation of a modulated excitation and a second modulation in the emission. An interferometry-like setup is used for calibration with the calibration arm blocked during the sample measurement.

**Figure 2.**
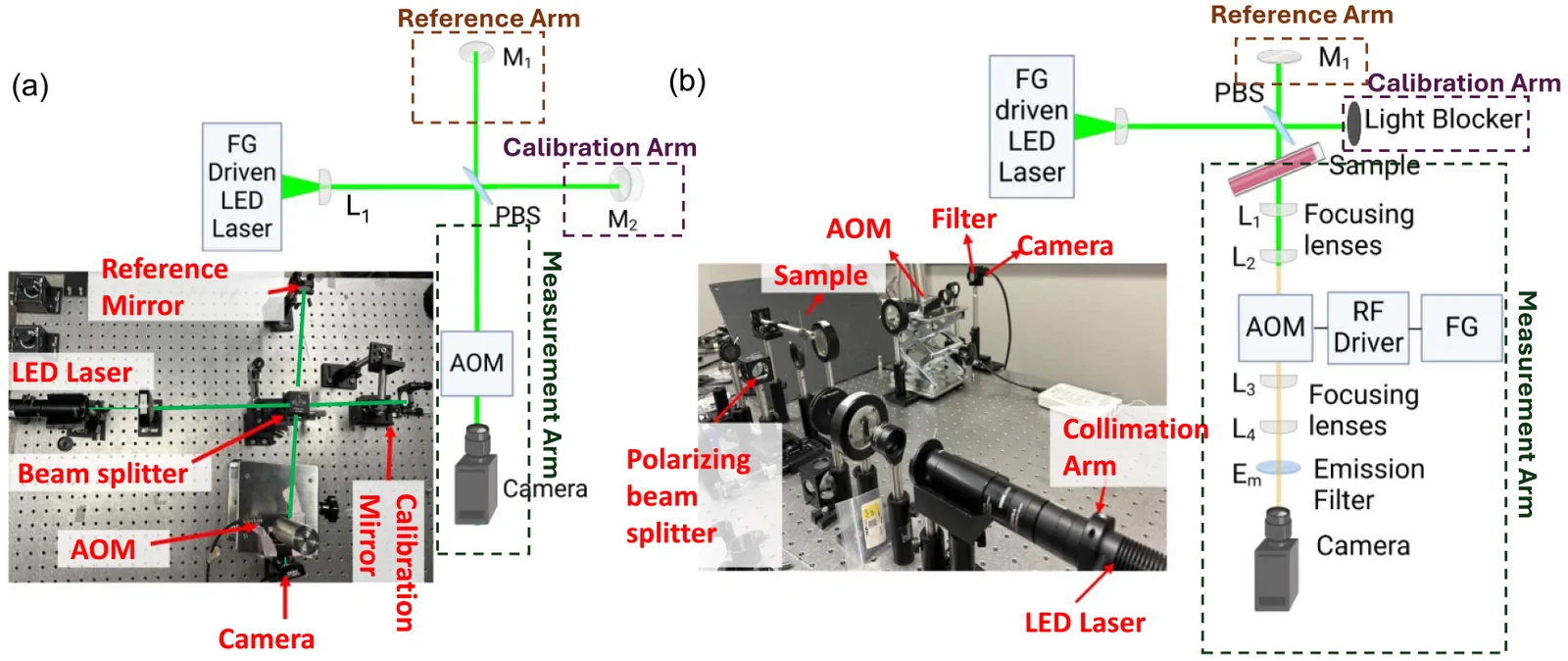
Schematic and experimental setup for transmission mode MAX-alif. (a) Setup for MAX-alif calibration. Intensity modulated light passes through a polarizing beam splitter (PBS) and is split to reference and calibration arms which have mirrors, one fixed (M1, reference) and one translated to different distances (M2, calibration). Light returning from the mirrors is then recombined by the PBS, passed through the AOM, and is detected by a camera. ($L_{1}$ denotes the collective set of lenses for collimating the illumination) (b) Experimental MAX-alif setup for imaging samples. Excitation light passes through the PBS to a reference arm with a mirror that reflects the light back through the PBS. The reflected light is combined with the emission light from the sample and focused through lens $L_{1}$ and $L_{2}$, passed through the AOM, focused by lens $L_{3}$ and $L_{4}$ and finally passed through an emission filter ($E_{m}$) to detection on a camera. FG = function generator. RF = Radiofrequency.

**Figure 3.**
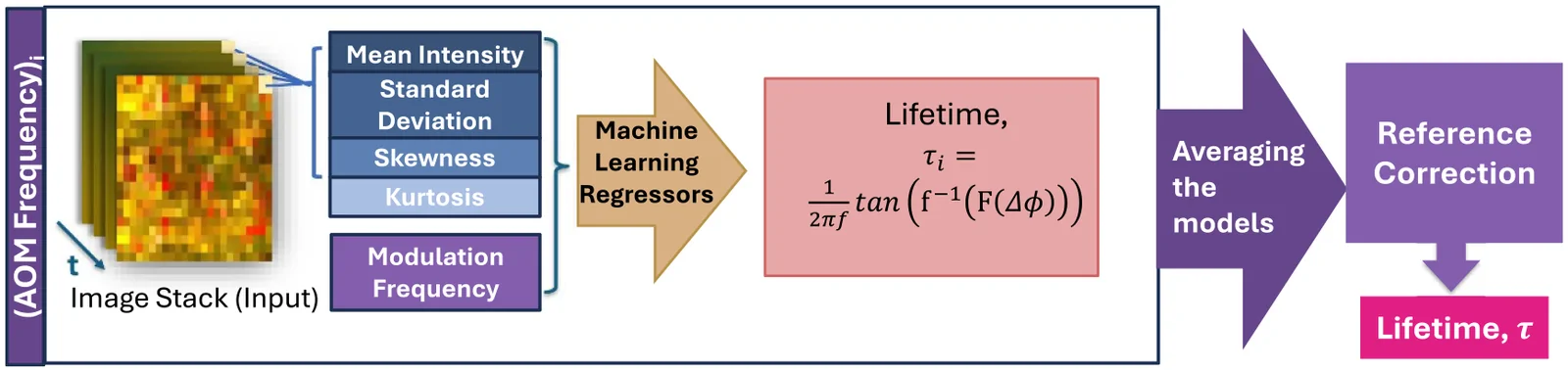
Algorithm for MAX-alif data analysis and regression model calibration process. First, the intensity statistical parameters (mean $\mu_{i}$, standard deviation $\sigma_{i}$, skewness $\gamma_{i}$, kurtosis $\kappa_{i}$, excitation modulation frequency $f_{\text{exc}}$ and emission modulation frequency $f_{\text{chopper}}$) are computed and used as features for a supervised regression fit. The lifetime is computed by the regressor and corrected for reference and dark noise.

**Figure 4.**
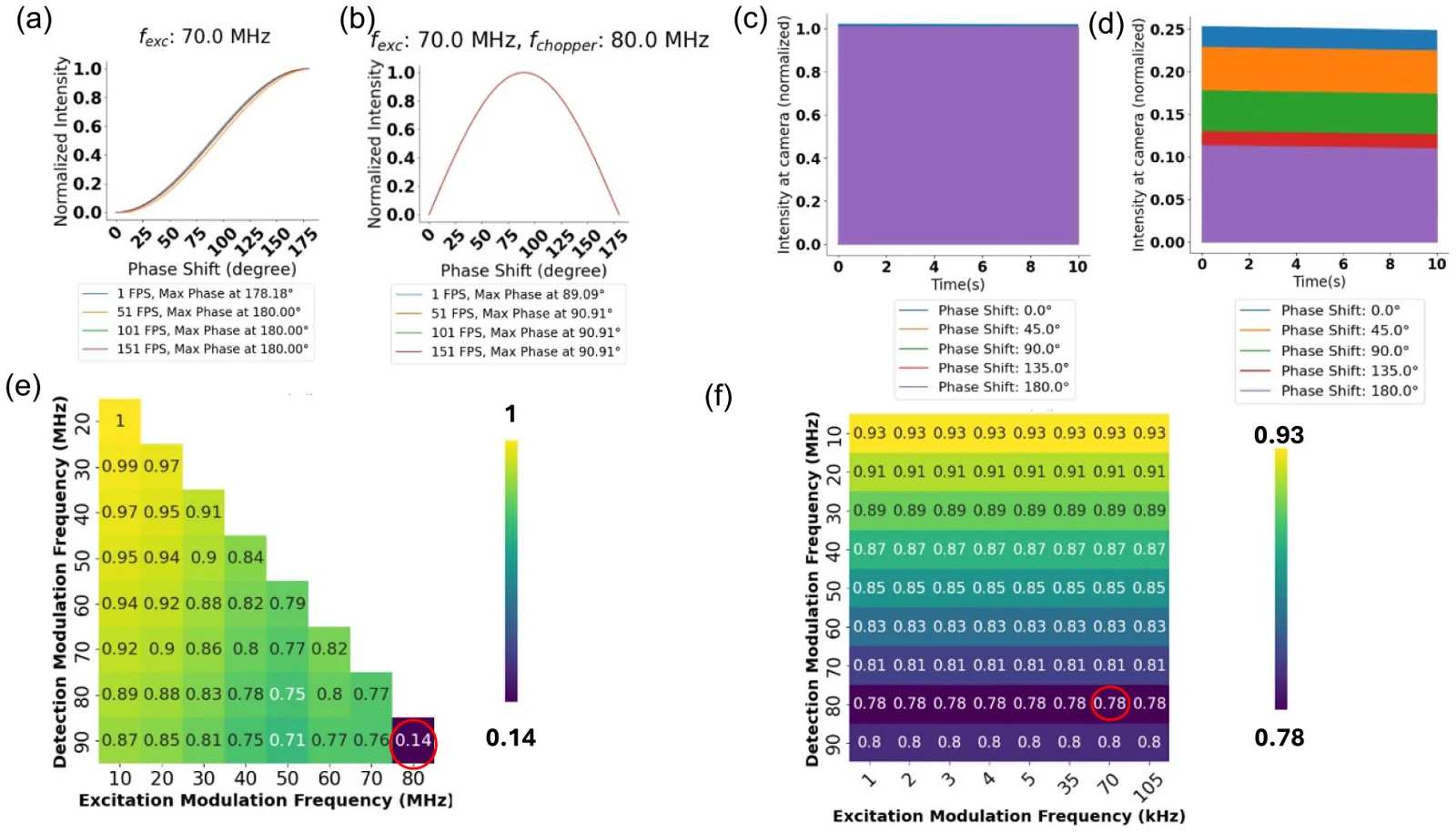
(a) Relationship between emission phase shift (with respect to excitation) and mean intensity of $I_{\text{camera},\text{unchopped}}$ for an excitation modulation frequency, $f_{\text{exc}}$, of 70 MHz and different camera frame rates with a constant 5 ms exposure time, (b) Relationship between emission phase shifts (with respect to excitation) and mean intensity of $I_{\text{camera},\text{chopped}}$ for an excitation modulation frequency, $f_{\text{exc}}$, of 70 MHz, an AOM frequency of 80 MHz, and different frame rates of the camera at 5 ms exposure time, (c) Simulations show the translation of lifetime induced phase shifts to intensity differences for the unchopped signal, $I_{\text{camera},\text{unchopped}}$, and all simulated phase shifts have nearly the same intensity. (d) Simulations for the translation of lifetime induced phase shifts to intensity differences for $I_{\text{camera},\text{chopped}}$ demonstrate different intensity values for different phase shifts. (e) Fluorescence regime error optimization map of equation (10) for different excitation light modulation frequencies and detection modulation frequencies. Lower values correspond to lower error. (f) Phosphorescence regime error optimization map of equation (10) for different excitation light modulation frequencies and detection modulation frequencies. Lower values correspond to lower error.

**Figure 5.**
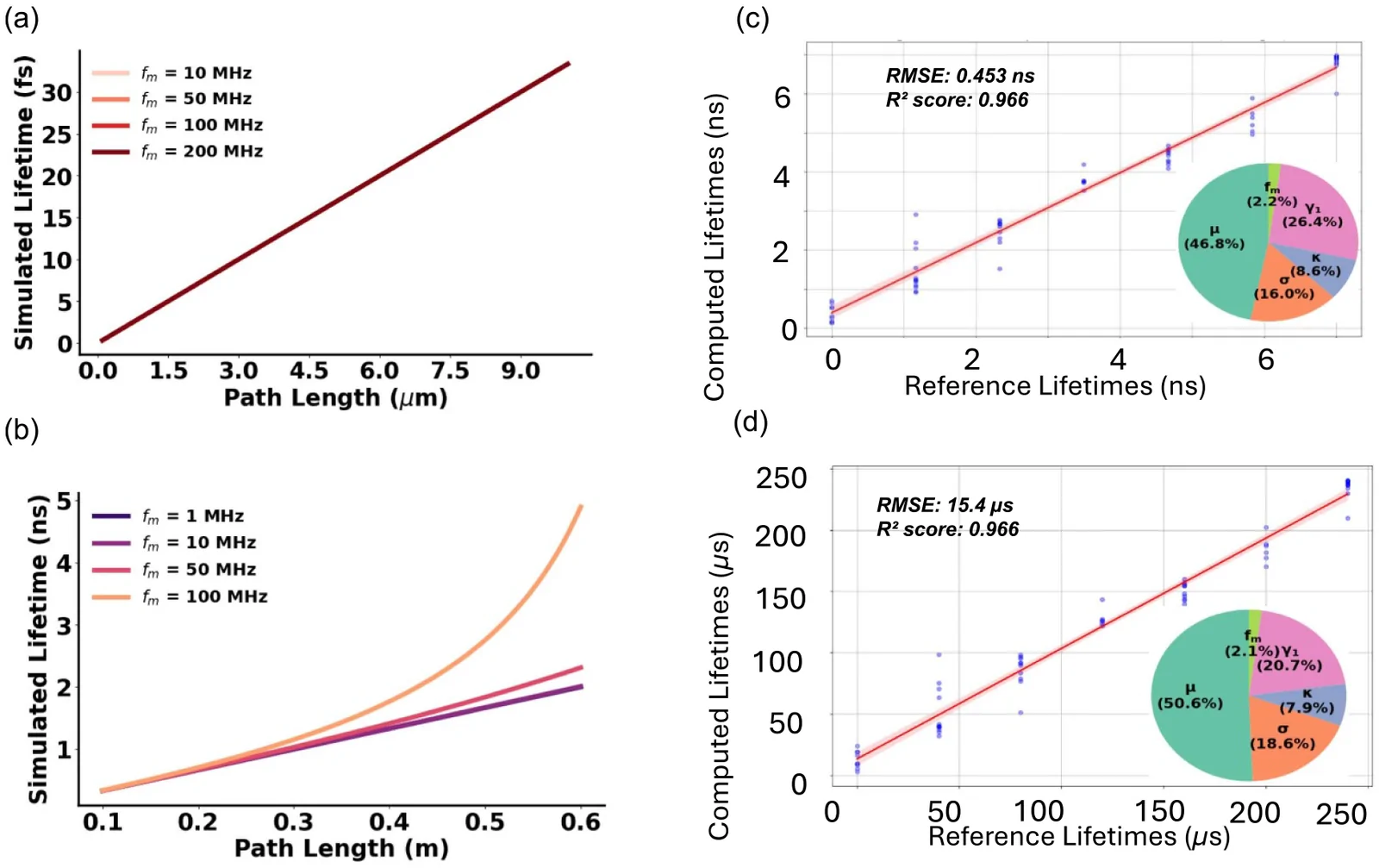
(a) Lifetime values calculated for path-length distances (Δx) 10 *μ*m to 100 *μ*m based on equation (3), (b) Lifetime values calculated for path-length distances from 0.1 m to 0.6 m based on equation (3), (c) Experimental MAX-alif calibration curve for computation of fluorescence lifetimes (inset- percentage importance of intensity features in the regression model), (d) Experimental MAX-alif calibration curve for computation of phosphorescence lifetimes (inset- percentage importance of intensity features in the regression model).

**Figure 6.**
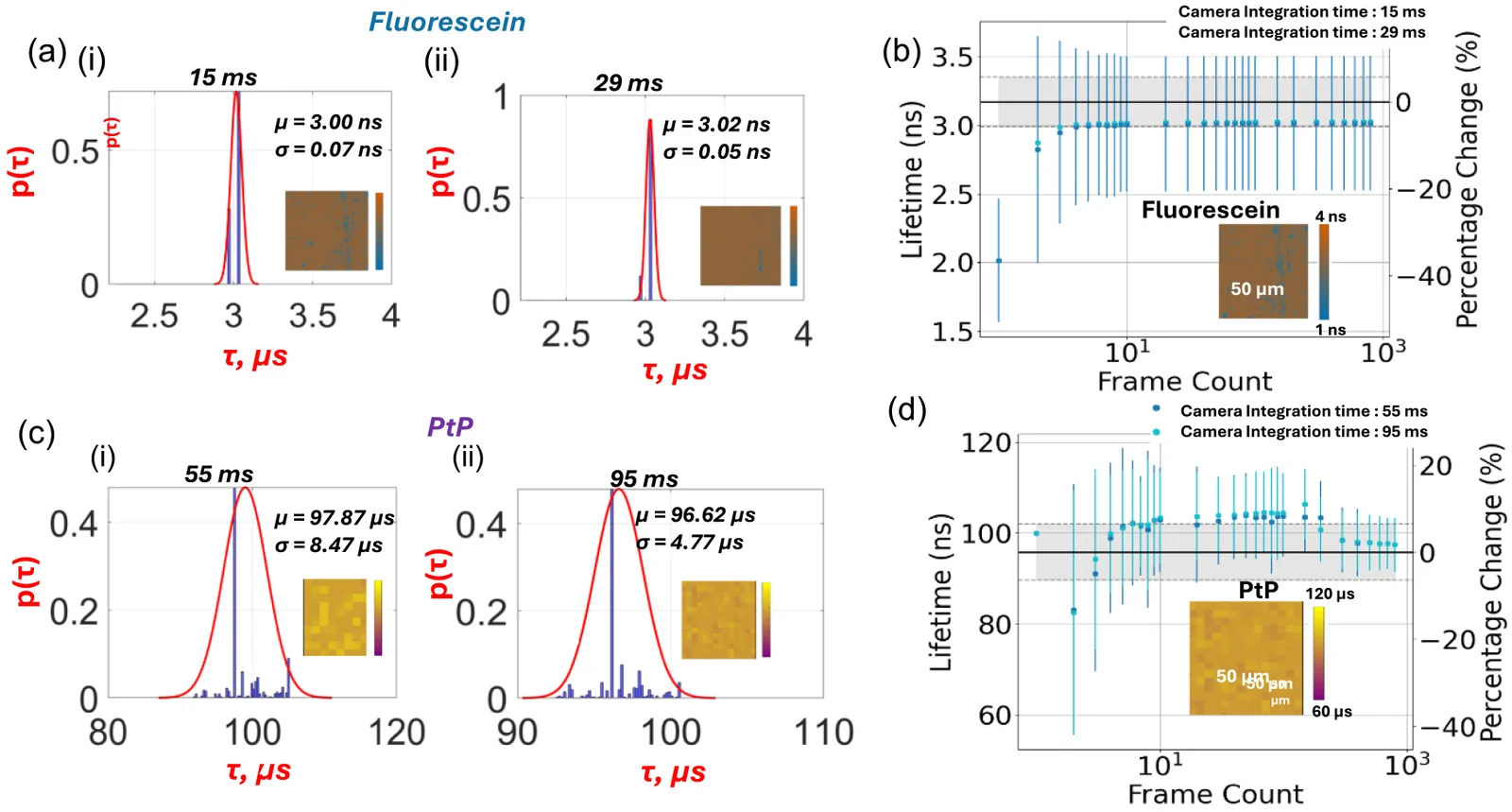
(a) Lifetime distribution and MAX-alif images of the fluorescein solution acquired at (i) 15 ms/34 FPS and (ii) 29 ms/34 FPS frame durations (scale bar represents 50 *μ*m), (b) MAX-alif computed lifetime values of fluorescein as a function of the number of image frames used to calculate the statistical features for inputs into the regression model for image stacks acquired at 15 ms/34 FPS and 29 ms/34 FPS. The horizontal black line and shaded box represent the mean and standard deviation of the TCSPC-measured value, 3.17 ns ± 0.18 ns. (c) Lifetime distribution and MAX-alif images of the PtP dye solution acquired at (i) 55 ms/18 FPS and (ii) 95 ms/10 FPS frame durations (scale bar represents 50 *μ*m), (d) MAX-alif computed lifetime values of fluorescein as a function of the number of image frames used to calculate the statistical features for inputs into the regression model for image stacks acquired at 55 ms/18 FPS and 95 ms/10 FPS. The horizontal black line and shaded box represent the mean and standard deviation of the TCSPC-measured value, 3.17 ns ± 0.18 ns.

**Figure 7.**
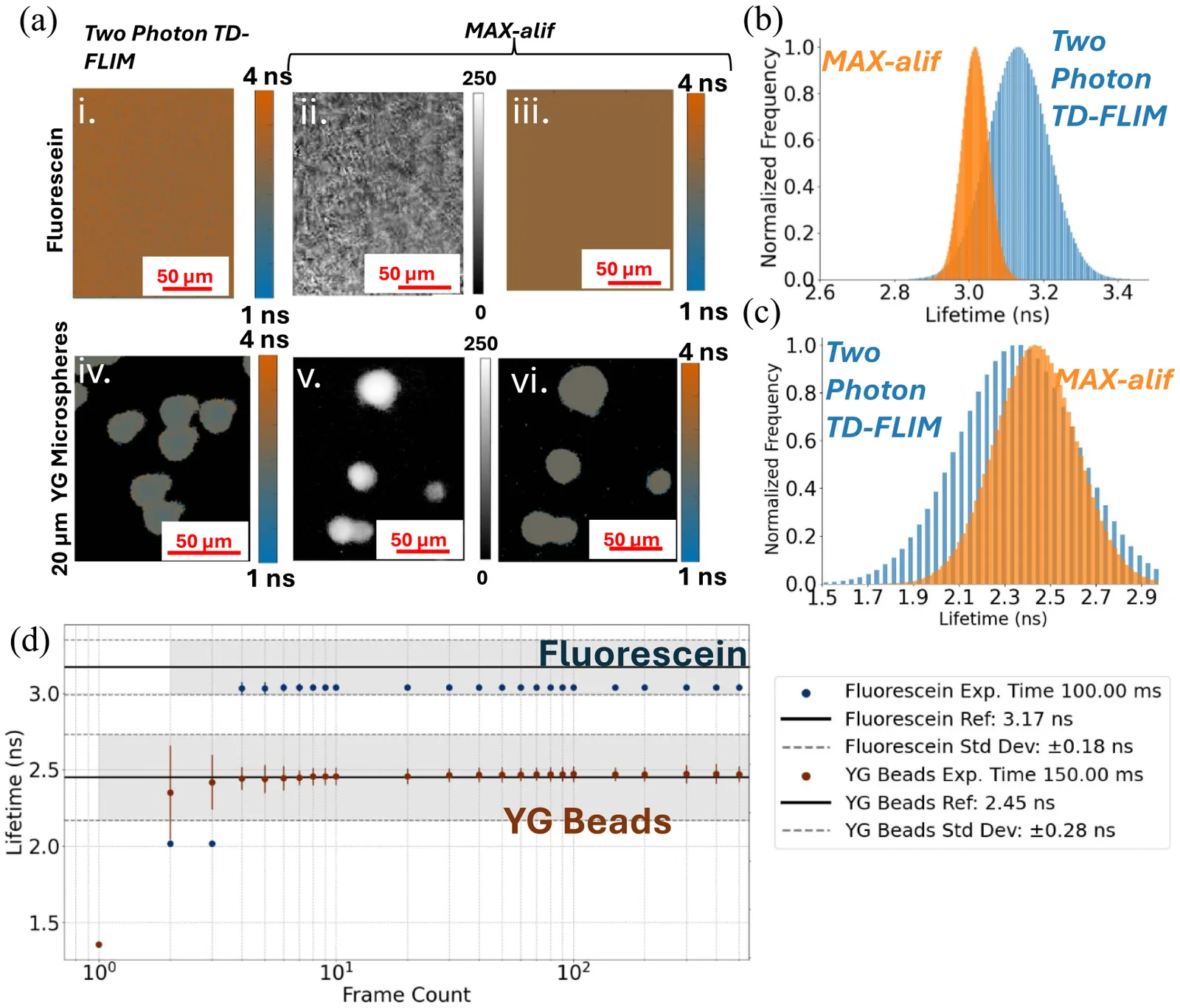
MAX-alif measured fluorescence lifetimes in a reflectance configuration. (a) Fluorescence Lifetime Images for fluorescein ((i)–(iii)), and 20 *μ*m-diameter YG microspheres ((iv)–(vi)) obtained using a two photon TCSPC FLIM system (i) and (iv) and MAX-alif (ii), (iii), (v) and (vi). (b) Distribution of lifetime values for pixels within the Fluorescein images acquired by both MAX-alif (orange) and TCSPC-FLIM (blue), (c) Distribution of lifetime values for pixels within the YG microsphere images acquired by MAX-alif (orange) and TCSPC-FLIM (blue), (d) Frame Rate Optimization study for the reflectance MAX-alif system images of fluorescein and YG microsphere samples.

## Data Availability

The data that support the findings of this study are available upon reasonable request from the authors. As a provisional patent application has been filed by the Texas A & M Engineering Experiment Station on behalf of the authors, the data cannot be made publicly available at this time.
