# Supplementary material for: Low-cost luminescence lifetime system for medical diagnostics
Source: JPhys Photonics. Author manuscript; Available in PMC 2026 Nov 7. (PMC13742190; doi:10.1088/2515-7647/ae15cf)
Supplement: Supplementary file 1 [file NIHMS2218484-supplement-Supplementary_Figures_and_Tables.docx]

Supplemental Information

Low-Cost Luminescence Lifetime System for Medical Diagnostics

Lakhvir Singh^1^, Melody Yeh^1^, Linghao Hu^1^, Brian Ko^1^, Michael J Mcshane^1,2,3^ and Alex J Walsh^1,*^

^1^Department of Biomedical Engineering, Texas A&M University, College Station, Texas, USA

^2^Department of Materials Science & Engineering, Texas A&M University, College Station, Texas, USA

^3^Center for Remote Health Technologies and Systems, Texas A&M University, College Station, Texas, USA

*walshaj@tamu.edu

1. Modeling MAX-alif

Following is a Table which illustrates the theoretical effect of wave interferometry and optical chopping on phase shift measurement for the luminescent signals for different cases of excitation and emission modulation frequencies.

| $\boldsymbol{f}_{\boldsymbol{exc}}$ | **Ideal** | **Unchopped** | **Chopped** |
| --- | --- | --- | --- |
| **Fluorescence Regime** | | | |
| 10  MHz |  |  |    |
| 20 MHz |  |  |      |
| 30 MHz |  |  |      |
| 40 MHz |  |  |         |
| 50 MHz |  |  |    |
| 60 MHz |  |  |  |
| 70 MHz |  |  |  |
| 80 MHz |  |  |  |
| **Phosphorescence Regime** | | | |
| 1 kHz |  |  |    |
| 2 kHz |  |  |   . |
| 3 kHz |  |  |        |
| 4 kHz |  |  |        |
| 5 kHz |  |  |      |
| 35 kHz |  |  |        |
| 70 kHz |  |  |  |
| 105 kHz |  |  |        |

Table 1. Relationship between Emission Phase Shifts (with respect to excitation) and their effect on mean intensity of the signal at different excitation and detection modulation frequencies and different frame rates of the camera.

Fig. S1 Area under curve (AUC) based optimization map of the optimization defined in ‘Optimization of Frequency Parameters’ subsubsection of the ‘Simulation Model’ subsection of the ‘Methods’ section to evaluate the maximum error for different excitation light modulation frequencies and AOM frequencies for fluorescence regime (a-d) and phosphorescence regime (e-h) at frame rates of 1, 51, 101 frames per second (FPS). Error is minimized for AUC = 1 (normalized). (d) Area under curve (AUC) based optimization map of Eq. \ref{cam_eq14} to evaluate the maximum error for different excitation light modulation frequencies and AOM frequencies for phosphorescence regime. Error is minimized for AUC = 1 (normalized).

2. Calibration of MAX-alif

Fig. S2 Feature Importance for Machine Learning Regressor (*XGBoost*) for each dataset corresponding to AOM frequency in case of (a) Fluorescence Regime, (b) Phosphorescence Regime.

Following are the figures which represent the model performance on the calibration for microsecond and nanosecond regime of lifetimes.

Fig. S3 (a) MAX-alif calibration curve for fluorescence regime, (b) Averaged feature importance plot for fluorescence regime, (c) Feature Importance for Machine Learning Regressor (*XGBoost*) for each dataset corresponding to AOM frequency in case of fluorescence regime, (d) MAX-alif calibration curve for phosphorescence regime, (e) Averaged feature importance plot for phosphorescence regime, (f) Feature Importance for Machine Learning Regressor (*XGBoost*) for each dataset corresponding to AOM frequency in case of phosphorescence regime.

3 Sample preparation

The dye platinum-meso-tetra(4-carboxylphenyl)porphine (PtP) from Frontier Scientific (Logan, UT) was used to evaluate MAX-alif accuracy for quantifying phosphorescent lifetimes. Dye molecules were then encapsulated within an oxygen-impermeable polymer nanoparticle to minimize oxygen quenching as previously described [25], where the oxygen-sensitive phosphor is encapsulated within a relatively oxygen-impermeable polymer bead. Tetrahydrofuran (THF), poly(sodium-4-styrenesulfonate) (PSS, average MW 70 kDa) ethylene glycol, and 2,2-dimethoxy-2-phenylacetophenone (DMPAP) were obtained from Sigma Aldrich. Acetone was obtained from Macron Fine Chemicals. Poly(vinylidene chloride-co-acrylonitrile) (PViCl-PAN), 2-hydroxyethyl methacrylate (HEMA, ophthalmic grade), and tetra(ethylene glycol) dimethacrylate (TEGDMA) were obtained from Polysciences, Inc. To encapsulate the porphyrin dye in polyacrylonitrile beads, 8.9 mL THF was added to 71.1 mL acetone in a glass beaker and stirred together. Next, 400 mg PViCl-PAN, 0.2 mg of PtTCPP, and 1 mL of 1 mg/mL PSS were added to the solution and mixed at a moderate rate until all solute molecules were dissolved. 240 mL of deionized water was then added to the solution dropwise. Then the beaker was covered with aluminum foil with holes for ventilation and left mixing overnight at 85 degrees Celsius to evaporate off solvents.

To purify the solution, 1 mL of 1 mg/mL PSS was added to the solution, which was then mixed for 1 hour. The solution was then centrifuged at 10,000 RCF for 8.5 minutes and the supernatant was removed. The samples were resuspended in deionized water. Then the sample was centrifuged at the same speed and time, the supernatant was removed, and the pellet was resuspended in 70% EtOH for two wash cycles. For the fourth and final wash cycle, the sample was resuspended in deionized water for storage.

For testing the nanosecond lifetimes, an aqueous solution of fluorescein (available commercially in powdered form, formula weight 332.32 Da) was prepared in water at a concentration of 0.1 mM.

    To demonstrate imaging on a micrometer scale of spatial dimensions, 20 µm YG microspheres were used. 10X of 0.0267% solution of YG microsphere was added into 1 mL of deionized and imaged on the MAX-alif system.

4 **Ground truth data for PtP dye**

Fig. S4. (a) Phosphorescence intensity decay curve for PtP dye fit to exponential decay model to calculate lifetime, (b) lifetime of PtP calculated by curve fit using different exclusion fractions, (c) reduced chi-square value corresponding to the curve fit for each exclusion fraction.

5 **MAX-alif in reflectance mode**

For calibration of the reflectance mode MAX-alif (Fig. S5 (a-b)), the system included a 450 nm collimated laser (CPS450 - Collimated Laser-Diode-Pumped DPSS) as a light source, which was followed by an acousto-optic modulator (Matsushita EFL-M150R01) to intensity modulate the light. The excitation light was expanded by a beam expander that included an Achromatic Doublet lens with 25 mm focal length (AC254-025-A Ø1"), which was followed by another Achromatic Doublet lens with 50 mm focal length (AC254-050-A-ML Ø1"). The direction of the expanded beam was altered by an inclined mirror to lead it through a 60:40 polarizing beam splitter (PBS), with 40% directed towards a mirror (M_1_) set at a reference path length (10 cm) and 60% to an upright objective. M_1_ was translated towards the PBS one step (10 μm) at a time for ten iterations to simulate a lifetime-induced change in phase shift via path length difference.

Fig. S5 (a) Imaging setup for reflectance mode of MAX-alif, (b) Testing resolution of MAX-alif using 1951 USAF resolution test chart. (c) Resolution characterization of the system using a USAF 1951 resolution target. The corresponding line intensity profile across the selected group was used to extract the edge spread function (ESF) and line spread function (LSF). The full-width at half maximum (FWHM) of the LSF was ~18 µm, representing the effective spatial resolution of the imaging system.

  The beam returning from mirror M_1_ was then contracted using an Achromatic Doublet lens with 100 mm focal length (AC508-100-A Ø2", and an Achromatic Doublet lens with 75 mm focal length (AC508-075-A Ø2"). The contracted beam then passed into another AOM with aperture of about 5 mm x 4 mm and center frequency of 40 MHz. The beam was expanded again by using the reverse combination of the 75 mm and 100 mm Achromatic Doublet lenses. Finally, the signal was imaged with a CMOS camera (Pixelink M-Series M12BM-CYL Microscopy Monochrome Camera with a maximum frame rate of 23.40 Hz and maximum pixel dimensions as 4,096 by 3,000). Both the AOMs were controlled through an RF driver and a function generator. For iteration of path lengths and modulation frequencies, 400-600 image frames were acquired with a fixed frame rate of 40 frames per second (FPS). Image stacks were acquired for both AOMs at the same frequency which was iterated from 1 MHz to 100 MHz at steps of 10 MHz. The collected calibration data was then used to generate and test regression models to relate the camera-measured intensity features with phase difference for lifetime calculation.

   For the imaging in the reflectance configuration, the path of excitation light to mirror M_1_ was blocked and the path of excitation light going to the objective was opened to capture the sample intensity which was filtered by a 500 nm ± 40 nm bandpass filter (Thor Labs FBH500-40) before the detection arm comprising beam contraction, the second AOM, beam expansion, and then focusing on the Pixelink camera (Fig S5 (a)). The laser power of the excitation light used was 4.3 mW and the laser beam dimensions were 3 mm by 1 mm. For each sample imaged, 600-800 image frames were acquired at 34 FPS. Reference image stacks were taken without illumination for the same number of frames at 34 FPS to determine artifacts due to the dark noise of the camera.

The calibrated lifetime response for the system is shown in Fig. S6.

Fig. S6. MAX-alif measured fluorescence lifetimes in a reflectance configuration. (a) Error optimization map of Eq. 10 to evaluate the maximum error for different excitation light modulation frequencies and detection modulation frequencies for the fluorescence regime with MHz modulation frequencies. Lower values correspond to lower error. (b) Calibration fit for the reflectance mode MAX-alif training data demonstrates a highly linear relationship between the reference lifetime and MAX-alif computed lifetime values for lifetimes ranging from 1-6 ns, for the reflectance system. (c) Feature importance analysis of the reflectance MAX-alif regression model.

**References**

[25] Ko BS, Pradhan R, McShane MJ. Phosphorescence Lifetime Imaging for Monitoring an Insertable Hydrogel Multianalyte Sensor. In: 2024 46th Annual International Conference of the IEEE Engineering in Medicine and Biology Society (EMBC). IEEE; 2024. p. 1-4.
